# Identifying large vessel occlusion at first glance in telemedicine

**DOI:** 10.1007/s00415-023-11775-2

**Published:** 2023-05-18

**Authors:** Nils Schröter, Antonia Weiller, Michel Rijntjes, Andreas Harloff, Horst Urbach, Juraj Kukolja, Jürgen Bardutzky, Cornelius Weiller, Lena-Alexandra Beume

**Affiliations:** 1 Department of Neurology and Clinical Neuroscience, Faculty of Medicine, Medical Center-University of Freiburg, University of Freiburg, Breisacher Str. 64, 79106 Freiburg, Germany; 2 Department of Neuroradiology, Faculty of Medicine, Medical Center-University of Freiburg, University of Freiburg, Breisacher Str. 64, 79106 Freiburg, Germany; 3 Faculty of Health, Witten/Herdecke University, Witten, Germany; 4 Department of Neurology and Clinical Neurophysiology, Helios University Hospital Wuppertal, Wuppertal, Germany

**Keywords:** Stroke, Head and/or gaze deviation, LVO, Telemedicine

## Abstract

**Background:**

Telemedicine has rapidly emerged as an important tool in emergency neurology. In particular, reliable biomarkers of large vessel occlusions (LVOs) are critically necessary in order to identify the need for in-hospital mechanical thrombectomy (MT). Based on pathophysiological factors, we propose that the presence of head and/or gaze deviation alone signifies cortical hypoperfusion and is therefore a highly sensitive marker for the presence of LVO.

**Methods:**

We retrospectively analyzed a cohort of 160 patients, examined via telemedicine and suspected to have had an acute stroke; this included patients with ischemic or hemorrhagic stroke, transient ischemic attack, and stroke mimics. An assessment of head and gaze deviation and NIHSS score evaluation was performed. In a second analysis, patients who only had ischemia in the anterior circulation (*n* = 110) were evaluated.

**Results:**

Head and/or gaze deviation alone was found to be a reliable marker of LVO (sensitivity: 0.66/specificity: 0.92), as well as a sound indicator for MT (0.82/0.91), in patients with suspected ischemic stroke. The performance of this indicator further improved when patients with ischemia in the anterior circulation only were assessed (LVO: 0.70/0.93; MT: 0.86/0.90). In both analyses, head and/or gaze deviation served as a better indicator for LVO or MT compared to the prevalence of motor deficits or aphasia. Of note, in patients who had ischemia in the anterior circulation, head and/or gaze deviation performed better than the NIHSS score as an indicator for MT.

**Conclusion:**

These findings confirm that the presence of head and/or gaze deviation serves as a reliable biomarker in stroke-based telemedicine for the diagnosis of LVO, as well as a strong indicator for MT. Furthermore, this marker is just as reliable as the NIHSS score but easier to assess. We therefore suggest that any stroke patient who displays head and/or gaze deviation should immediately be scheduled for vessel imaging and subsequently transported to a MT-competent center.

## Introduction

The rapidly evolving application of telemedicine in acute stroke can serve as an important basis for identifying therapeutic indications such as intravenous thrombolysis (rtPA) [1] and mechanical thrombectomy (MT) [2, 3] in patients with large vessel occlusion (LVO) [4]. Since the therapeutic success of these procedures is strongly dependent on time, an early, accurate decision-making process is essential in order to enable timely transfer of the patient to a MT-competent center for appropriate stroke-oriented imaging and therapy.

We have previously shown that neuropsychological deficits such as aphasia and neglect are reliable indicators for LVO and MT in the emergency room, and that they are more sensitive and specific than pure motor symptoms [5]. This is because neuropsychological deficits can only arise from cortical hypoperfusion (in the case of LVO) due to insufficient leptomeningeal blood flow, whereas motor deficits can be caused by lesions at various sites including the internal capsule and brainstem [6, 7]. Head and/or gaze deviation is a sign of neglect in cases of hypoperfusion in the parietal/temporal lobes or inferior frontal gyrus [8–10], or can alternatively be attributed to gaze palsy arising from frontal eye field dysfunction [11]. Therefore, head/gaze-deviation are associated with the area of the cerebral cortex that is supplied by the middle cerebral artery, the main target of thrombectomy in LVO. In our previous study, we identified the presence of neglect alone as a strong predictor of LVO [5]. However, diagnosing visual neglect or extinction based on the NIHSS (National Institutes of Health Stroke Scale) requires neurological expertise.

Therefore, in the present study we further simplified the clinical assessment procedure by investigating head and gaze deviation as the primary characteristic of visual neglect, since this phenomenon is easily assessed and can be seen “at first glance” via teleconsultation. We hypothesized that the presence of gaze- and/or head deviation alone would serve as a strong, easily applicable biomarker of LVO, as well as a reliable indicator of MT that can be effectively applied in telestroke medicine.

## Methods

### Participants and clinical assessment

Our telemedicine network FRITS (Freiburger Telemedizin für Schlaganfall-behandlung) includes 11 hospitals with local stroke units in Southwest Germany, all within a 220-km radius of Freiburg. The present study is a retrospective analysis of clinical and imaging data from consecutive patients who were referred for treatment at a local stroke unit at one of the above-mentioned 11 hospitals between 01 August 2018 and 08 July 2019 via our telemedicine network (based at the Department of Neurology and Clinical Neuroscience, Medical Center, Freiburg). All patients presented at one of the participating centers with a suspected diagnosis of stroke or with new or worsened neurological deficits received a tele-neurological consultation. In accordance with the recent recommendation for the treatment window of MT in acute cerebral ischemia, all patients who presented to us within 24 h of symptom onset (or were “last seen well” 24 h before presentation) were included.

**Fig. 1:**
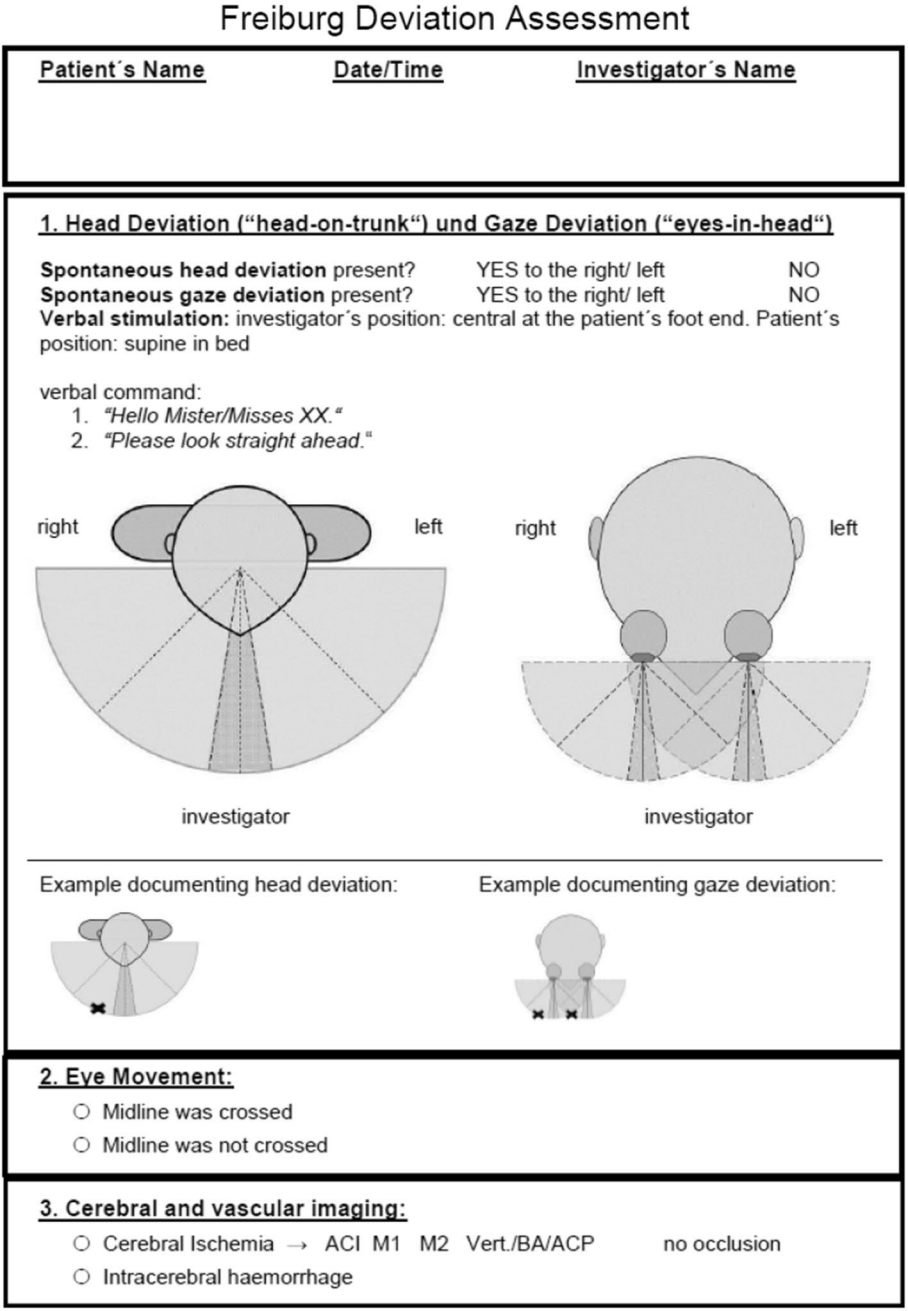
The Freiburg Deviation Assessment (FDA)

In the preclinical setting, ambulance staff are given a basic introduction to neurological examination as part of their training. The FAST (Face, Arm, Speech, Time) method is used specifically to detect a stroke in the preclinical setting. Primary patient care was provided by non-board-certified internists on site, who were trained by J.B. to perform the neurological examination. The examination that took place via telemedicine was performed by a board-certified neurologist (A.H.). Recent studies suggest that NIHSS score assessment performed via video conference is comparable in terms of feasibility and reliability to bedside NIHSS assessment when a stroke specialist is not available on site [12]. The total NIHSS score was therefore assessed during the teleconsultation and the following items were evaluated: presence of hemiparesis in an upper or lower extremity (drift of any severity) and the presence of aphasia. For the evaluation of aphasia, patients were asked to name an object shown to them (a ballpoint pen). Attention was also paid to spontaneous speech and the ability to follow verbal commands during the routine interaction. Aphasia was deemed to be present when the patient did not identify the object or follow the verbal command, or in the case of severe loss of fluency (but not anarthria). Head and/or gaze deviation was diagnosed when spontaneous deviation of head and/or gaze position was present, and/or horizontal eye movement did not cross the midline, even when the attending physician applied verbal or tactile stimuli. Documentation was based on the Freiburg Deviation Assessment “FDA” (Fig. 1), a simple standardized documentation tool for identifying signs of head and gaze deviation.

**Fig. 2:**
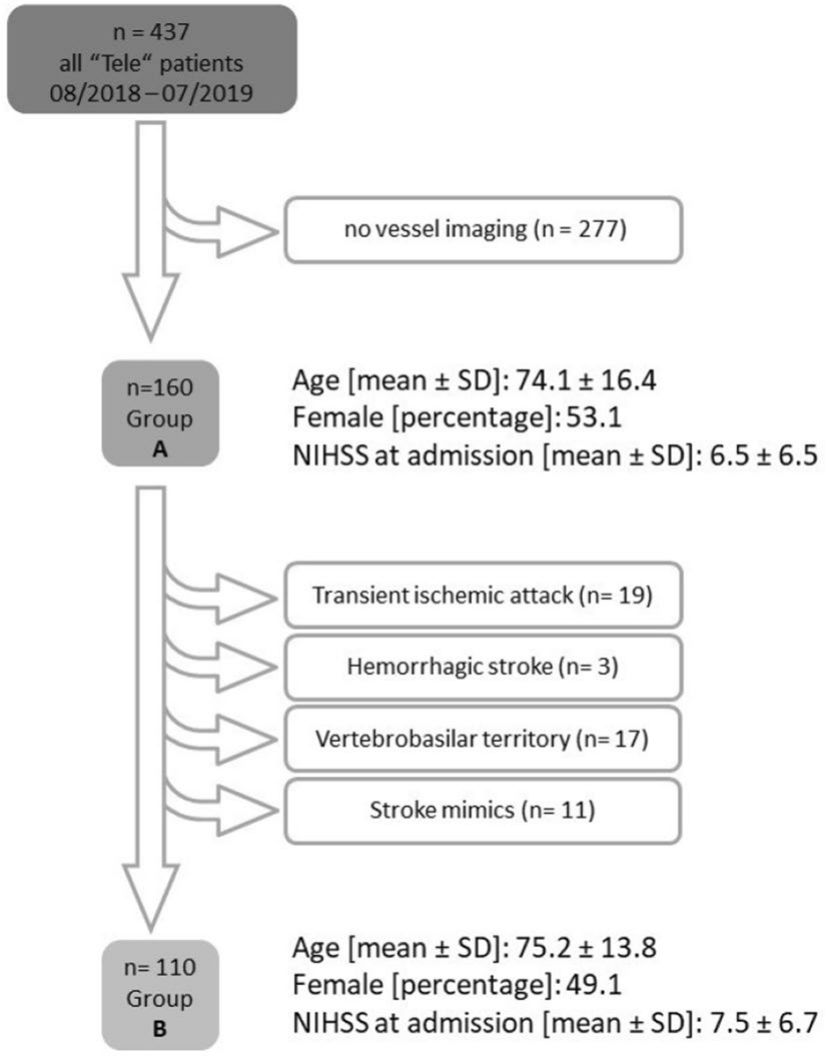
Flowchart describing the allocation of the stroke patient cohort into Group A (*n* = 160) and Group B (*n* = 110). *SD* standard deviation

A total of 3701 patients were consecutively examined via telemedicine within a 12-month period. Due to data protection restrictions, standardized documentation of gaze and head deviation was only possible during the day, since night-time telemedical assessment was performed externally and FDA documentation was not permitted. Thus, 437 patients underwent structured FDA documentation and 160 of these patients had vascular imaging either via computed tomography (CT) angiography or magnetic resonance tomography (MRT) angiography (Group A, *n* = 160). In the remaining cases, vascular imaging was not performed because of demarcated infarcts that fully explained the symptoms, or due to intracerebral hemorrhages or transient deficits. In a further step, patients with either cerebral hemorrhage (*n* = 3), marked improvement of symptoms with complete remission during or immediately after cerebral imaging (TIA, *n* = 19), vertebrobasilar ischemia (*n* = 17) and stroke mimics (*n* = 11) were excluded from group A, resulting in Group B (*n* = 110) (Fig. 2). This second-step analysis was conducted to ensure comparability of our results with those of previous studies in which scores were tested on similar patient groups [13–18].

One neuroradiologist (H.U.) and one vascular neurologist (A.H.) independently assessed the CT or MR angiography findings. LVO was defined as the occlusion of either the internal carotid artery, the middle cerebral artery (M1) or the proximal segments of M2 on either side.

### Statistical analysis

Statistical analysis was performed with R software (version 4.0.3, http://www.R-project.org). Sensitivity, specificity, and positive/negative predictive values for motor deficits and neuropsychological deficits (i.e., aphasia, head or gaze deviation) were evaluated in patients with LVO and MT. A receiver operating characteristic (ROC) analysis was employed to assess the diagnostic performance of the NIHSS total score in patients with “LVO” versus “no LVO”. Patients with or without MT versus were compared using a means of the area under the ROC curve (AUC) analysis [19]. The optimal cut-off value was determined by Youden's index.

### Ethical compliance

This retrospective analysis was approved by the local institutional review board of the University Hospital Freiburg (EK 21-1334) and carried out in accordance with the Declaration of Helsinki and its later amendments.

### Data availability

The data supporting the findings of this study are available upon reasonable request and subject to approval from the local ethics committee.

## Results

### Patient characteristics

A detailed description of patient characteristics is provided in Table 1. The mean age (± standard deviation, SD) of patients in Group A was 73.5 ± 14.5 years, and the mean NIHSS score was 6.5 ± 6.5. LVO was detected in 32/160 cases, whereby 22 cases received MT. The contraindications for MT were as follows: extensive infarct demarcation corresponding to the hypoperfused area (*n* = 4), poor general condition of the patient (*n* = 2) or poor recovery following bridging treatment of the open vessel after transportation to the MT-competent center (*n* = 4). In Group B, the mean age was 75.2 ± 13.7 years and the mean NIHSS score was 7.5 ± 6.7. LVO was detected in 30/110 cases, whereby 21 received MT.

**Table 1:** Demographic characteristics and basic clinical data

Group A	Overall	Group B	Overall
	(*N* = 160)		(*N* = 110)
*Age*			
Mean (SD)	73.5 (14.5)	Mean (SD)	75.2 (13.8)
*Sex*			
Male	75 (46.9%)	Male	56 (50.9%)
Female	85 (53.1%)	Female	54 (49.1%)
*Large vessel occlusion*			
No	128 (80.0%)	No	80 (72.7%)
Yes	32 (20.0%)	Yes	30 (27.3%)
*Mechanical thrombectomy*			
No	138 (86.3%)	No	89 (80.9%)
Yes	22 (13.8%)	Yes	21 (19.1%)
*Paresis*			
No	74 (46.3%)	No	42 (38.2%)
Yes	86 (53.8%)	Yes	68 (61.8%)
*Aphasia*			
No	113 (70.6%)	No	73 (66.4%)
Yes	47 (29.4%)	Yes	37 (33.6%)
*Head and/or gaze deviation*			
No	129 (80.6%)	No	83 (75.5%)
Yes	31 (19.4%)	Yes	27 (24.5%)
*NIHSS-Score*			
Mean (SD)	6.48 (6.54)	Mean (SD)	7.51 (6.71)

### Clinical assessment

In *Group A*, the prevalence of head and/or gaze deviation alone showed for LVO a sensitivity level (SEN) of 0.66, a specificity level (SPE) of 0.92, a positive predictive value (PPV) of 0.68, and a negative predictive value (NPV) of 0.9. Head and/or gaze deviation predicted a subsequent indication for MT with a SEN of 0.82, a SPE of 0.91, a PPV of 0.58, and a PPV of 0.97. In comparison to head and/or gaze deviation alone, motor symptoms alone were more sensitive for LVO (0.81) and MT (0.86), albeit less specific (0.53; 0.51), while the presence of aphasia alone was both less sensitive (0.56 for LVO, 0.64 for MT) and less specific (0.77 for LVO, 0.76 for MT). While the combination of cortical deficits, i.e. head and/or gaze deviation and aphasia, was more sensitive (0.81 for LVO; 0.91 for MT), it was also shown to be less specific (0.74 for LVO; 0.72 for MT).

Statistical analysis revealed that the highest level of sensitivity was achieved when either cortical deficits or motor deficits were present (0.91 for LVO, 0.95 for MT), whereas the level of specificity dropped to 0.41 for LVO and 0.39 for MT (Table 2).

**Table 2:** Diagnostic performance of cortical and motor deficits and their combination in group A

Item	SEN LVO/MT	SPE LVO/MT	PPV LVO/MT	NPV LVO/MT	ACC LVO/MT
*Group A*					
Paresis	0.81/0.86	0.53/0.51	0.3/0.22	0.92/0.96	0.59/0.56
Aphasia	0.56/0.64	0.77 0.76	0.38/0.3	0.88/0.93	0.73/0.74
Head and/or gaze deviation	0.66/0.82	0.92/0.91	0.68/0.58	0.91/0.97	0.87/0.89
Aphasia or head and/or gaze deviation	0.81/0.91	0.74/0.72	0.44/0.34	0.94/0.98	0.76/0.74
Aphasia and head and/or gaze deviation	0.41/0.55	0.95/0.95	0.68/0.63	0.87/0.93	0.84/0.89
Paresis or aphasia	0.91/0.95	0.41/0.4	0.28/0.2	0.95/0.98	0.81/0.48
Paresis and aphasia	0.47/0.55	0.89/0.88	0.52/0.41	0.87/0.92	0.81/0.83
Paresis or head and/or gaze deviation	0.84/0.91	0.52/0.5	0.3/0.22	0.93/0.97	0.58/0.56
Paresis and head and/or gaze deviation	0.63/0.77	0.94/0.92	0.71/0.51	0.91/0.96	0.88/0.9
Paresis or cortical	0.91/0.95	0.41/0.39	0.28/0.2	0.95/0.98	0.51/0.47
Paresis and cortical	0.72/0.82	0.87/0.84	0.58/0.45	0.93/0.97	0.84/0.84

*LVO* large vessel occlusion; *MT* mechanical thrombectomy; *SEN* sensitivity; *SPE* specificity; *PPV* positive predictive value; *NPV* negative predictive value; *ACC* accuracy

The same analysis was performed in *Group B*, which included all patients with an ischemic stroke in the anterior circulation (Table 3). In this case, head and/or gaze deviation alone was associated with a higher mean sensitivity score compared to that in Group A (0.70 for LVO, 0.86 for MT); when compared to motor deficits, it had an equivalent level of sensitivity (0.83 for LVO, 0.86 for MT) but a still much higher level of specificity (0.93 for LVO, 0.90 for MT versus 0.46 for LVO, 0.44 for MT). Head and/or gaze deviation was again a more sensitive and specific predictor of LVO and MT indication than aphasia alone (aphasia: 0.56 for LVO, 0.64 for MT versus head/gaze deviation: 0.77 for LVO, 0.76 for MT). The presence of any type of cortical sign was more sensitive (0.83 for LVO, 0.90 for MT) and similarly specific (0.73 for LVO, 0.69 for MT) in comparison to any symptom assessed individually. The highest degree of sensitivity was once again reached in the presence of either cortical or motor deficits (0.93 for LVO, 0.95 for MT). For a direct comparison between head and/or gaze deviation and NIHSS see Table 4.

**Table 3:** Diagnostic performance of cortical and motor deficits and their combination in group B

Item	SEN LVO/MT	SPE LVO/MT	PPV LVO/MT	NPV LVO/MT	ACC LVO/MT
*Group B*					
Paresis	0.83/0.86	0.46/0.44	0.37/0.26	0.88/0.93	0.56/0.52
Aphasia	0.57/0.62	0.75/0.73	0.46/0.35	0.82/0.89	0.7/0.71
Head and/or gaze deviation	0.7/0.86	0.93/0.9	0.78/0.67	0.89/0.96	0.86/0.89
Aphasia or head and/or gaze deviation	0.83/0.9	0.73/0.69	0.53/0.4	0.92/0.97	0.75/0.73
Aphasia and head and/or gaze deviation	0.43/0.57	0.95/0.94	0.76/0.71	0.81/0.9	0.81/0.87
Paresis or aphasia	0.93/0.95	0.34/0.31	0.35/0.25	0.93/0.97	0.5/0.44
Paresis and aphasia	0.47/0.52	0.88/0.85	0.58/0.46	0.81/0.88	0.76/0.79
Paresis or head and/or gaze deviation	0.87/0.9	0.44/0.42	0.36/0.27	0.9/0.95	0.55/0.51
Paresis and head and/or gaze deviation	0.67/0.81	0.95/0.92	0.83/0.71	0.88/0.95	0.87/0.9
Paresis or cortical	0.93/0.95	0.33/0.3	0.34/0.24	0.93/0.96	0.49/0.43
Paresis and cortical	0.73/0.81	0.86/0.82	0.67/0.52	0.9/0.95	0.83/0.82

*LVO* large vessel occlusion; *MT* mechanical thrombectomy; *SEN* sensitivity; *SPE* specificity; *PPV* positive predictive value; *NPV* negative predictive value; *ACC* accuracy

**Table 4:** Direct comparison of the diagnostic performance of Head and/or gaze Deviation and NIHSS

		Head and/or gaze Deviation		NIHSS		ROC-AUC
		SEN	SPE	SEN	SPE	
Group A	LVO	0.66	0.92	0.84	0.76	0.84
	MT	0.82	0.91	0.91	0.72	0.88
Group B	LVO	0.70	0.93	0.87	0.71	0.84
	MT	0.86	0.90	0.71	0.85	0.86

*SEN* sensitivity; *SPE* specificity; *ROC-AUC* receiver operating characteristics area under the curve

### ROC-AUC

In Group A, ROC analysis indicated that a cut-off NIHSS score of 6.5 was sufficient to discriminate between the presence and absence of LVO, with a sensitivity level of 0.84 and a specificity level of 0.76 (ROC-AUC = 0.84). Furthermore, this cut-off score could predict an indication for MT with a sensitivity level of 0.91 and specificity level of 0.72 (ROC-AUC = 0.88).

**Fig. 3:**
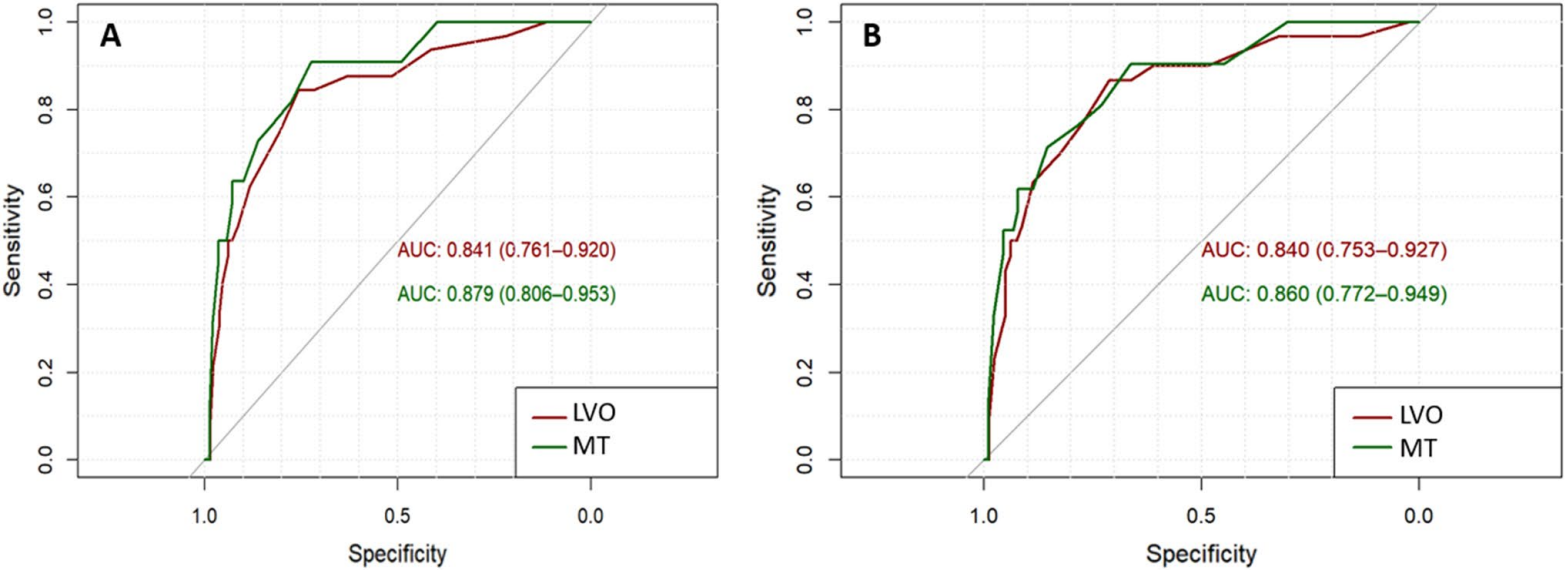
ROC curves illustrating the validity of the NIHSS score in Group A (left) and Group B (right) in predicting both large vessel occlusion (LVO, red) and the indication for mechanical thrombectomy (MT**,** green). AUC, area under the curve

In Group B, a cut-off NIHSS score of 6.5 could discriminate between the presence and absence of LVO with a sensitivity level of 0.87 and specificity level of 0.71 (ROC-AUC = 0.84), while a cut-off NIHSS score of 9.5 best discriminated between MT indication versus no MT indication, with a sensitivity level of 0.71 and a specificity level of 0.85 (ROC-AUC = 0.86) (see Fig. 3).

## Discussion

Our data show that the cortical deficits of head and/or gaze deviation alone are moderately sensitive but highly specific markers for LVO presence and MT indication in the acute tele-stroke setting. The levels of sensitivity and specificity increase when head and/or gaze deviation and aphasia are evaluated together, with the combination of any cortical symptoms being more specific than one cortical symptom alone. Statistical analysis further revealed that the use of motor symptoms as a predictor of LVO presence and MT indication leads to the highest sensitivity scores but is also associated with a very low level of specificity. In contrast, the combination of cortical symptoms AND motor symptoms serves as a predictor with the highest specificity rate.

In comparison to our previous study, the sensitivity of a head and/or gaze deviation examination in a teleconsultation setting is slightly inferior to that of a bedside assessment of neglect (sensitivity of detecting LVO at bedside versus teleconsultation: 76% vs 70%; sensitivity of detecting MT at bedside versus teleconsultation: 88% vs 86%) [5]. This is likely explained by the fact that in our first publication, neglect was evaluated and examined by experienced neurologists, while in this study, no specific (or supplementary) training for recognizing head and/or gaze deviation via camera was necessary. However, head and gaze deviation appears to have a remarkable degree of specificity in the diagnosis of LVO. As a single diagnostic marker for LVO, the presence of this cortical deficit displays an equal level of diagnostic performance compared to either that of the total NIHSS score, or to the more elaborate scores presented in Table 4 [13, 14]. It is interesting to note that in the most clinically relevant subgroup of patients with ischemia in the anterior circulation, head and/or gaze deviation had a higher level of sensitivity and specificity than the NIHSS in predicting an indication for MT.

The anatomical basis of this high specificity for gaze or head deviation can be explained as follows: in the context of neglect, gaze or head deviation are typical signs of reduced exploration within the contralesional space [20], and lesions in the temporal or parietal lobe in both hemispheres, as well as lesions in the inferior frontal gyrus have been associated with the formation of neglect [8–10, 21]. In addition, ischemia in the frontal eye field, which is located above the inferior frontal sulcus, leads to gaze deviation [11]. Thus, gaze or head deviation is indicative of a functional disturbance in any of the three cerebral lobes typically supplied by the M1; namely the frontal, parietal and temporal lobes. Therefore, an M1 or a proximal M2 occlusion, which nowadays serves as a typical indication for MT, causes hypoperfusion in the area of the cerebral cortex that is associated with either neglect or dysfunction of the frontal eye field; this is turn leads to head or gaze deviation, respectively. It is also worth noting that the occurrence of neglect is very similar in right and left hemispheric strokes in the acute phase [10].

As mentioned above, motor deficits such as arm paresis can occur after an even more focal lesion (e.g. “hand knob” in M1), or following a lacunar infarction within the motor pathway, and are thus not particularly specific to LVO. Isolated hemiparesis is a typical lacunar syndrome, arguing against LVO [22].

Head and gaze deviation can also be a sign of seizure occurrence, with the direction of deviation typically being opposite to paresis. However, ipsilateral gaze deviation is also observed in approximately 10% of seizure cases, mainly in temporal foci [23].

This retrospective analysis of our data has several limitations. Firstly, the decision to consult a neurologist from the telestroke network already implies a preselection bias. Secondly, all patients were seen by vascular neurologists, therefore, it is unclear whether our findings are applicable to less experienced physicians or those from other disciplines. However, since the detection of head and/or gaze deviation is a simple assessment that requires little clinical experience, we assume that the results can also be transferred to trainee neurologists as well as other medical professionals. This is especially relevant as most telestroke consultations are performed out of hours, like in this study, when a neurologist is not available in a timely manner and staffing levels are thin increasing the demand of an easy to apply first-glance biomarker for LVO.

We propose that these findings be used as a screening biomarker in the preclinical setting in order to be able to proceed without delay to the nearest neurovascular center in cases of suspected LVO. However, the use of head and gaze deviation as a biomarker in a preclinical emergency setting should first be validated in prospective studies. It must be emphasized that the presence of head and/or gaze deviation is indicative of, but the absence of head and/or gaze deviation does not exclude LVO due to the relatively low sensitivity, so that the absence of head and/or gaze deviation alone is not an indication against vascular imaging or MT.

These data confirm that the phenomenon of head and gaze deviation serves as a reliable biomarker not only for the diagnosis of LVO but also as a predictor of the need for MT in stroke-based telemedicine. These neurological signs are easy to evaluate and should therefore provide a cornerstone for clinical assessment. We therefore suggest that any stroke patient who displays head and/or gaze deviation should immediately be scheduled for vessel imaging and transport to a MT-competent center.
